# Syncope as the initial presentation of pulmonary embolism in two patients with hepatocellular carcinoma: Two case reports and literature review

**DOI:** 10.1097/MD.0000000000027211

**Published:** 2021-09-24

**Authors:** Dayong Deng, Haidi Wu, Huafang Wei, Zikai Song, Yang Yu, Chongyin Zhang, Lei Yang

**Affiliations:** a Department of Radiology, Jilin Provincial Cancer Hospital, Changchun, China; b Department of Cardiology, The First Hospital of Jilin University, Changchun, China.; c Department of Internal Medicine-1, Jilin Provincial Cancer Hospital, Changchun, China.

**Keywords:** hepatocellular carcinoma, pulmonary embolism, syncope

## Abstract

**Rationale:**

Pulmonary embolism (PE) has diverse clinical manifestations and syncope might be the first or only symptom of PE. Tumor disease usually presents with symptoms associated with the primary site, however, PE may be the first manifestation of occult tumors.

**Patient concerns:**

Here, we report 2 patients admitted to our hospital because of syncope. One patient had a chronic hepatitis B history of more than 20 years and the other patient had chronic heavy drinking for many years. Neither patient had been diagnosed with neoplastic disease before admission.

**Diagnoses:**

Clinical examinations, including laboratory tests and imaging tests upon admission demonstrated PE resulting in syncope. Furthermore, malignant hepatocellular carcinoma (HCC), inferior vena cava, and right atrium tumor thrombus were diagnosed.

**Interventions:**

Thrombolysis and anti-coagulation therapy were performed immediately after the diagnosis of PE. Twenty-seven HCC patients with PE in 27 articles from 1962 to 2020 in the PubMed database were reviewed.

**Outcomes:**

The improvement was achieved that no syncope recurred after treatment of PE. The oxygen partial pressure increased and the D-dimer level decreased. The clinical characteristics of 27 HCC patients with PE were summarized and analyzed.

**Lessons:**

It is important for clinicians to be aware that occult carcinoma might be a reason for patients with PE presenting with syncope. If PE cannot be explained by common causes, such as our patient, and HCC should be highly suspected when inferior vena cava and right atrial mass are found on imaging tests.

## 1 Introduction

Venous thrombus embolism (VTE) involves the formation of deep venous thrombosis (DVT) and pulmonary embolism (PE), which are common complications of cancer. Cancer frequently develops VTE, possibly due to increased platelet activation by tumor-derived pro-coagulant proteins, overexpression of tissue factors, and overproduction of inflammatory cytokines.^[1]^ The incidence of VTE in cancer is 4% to 20%,^[2]^ and it is also one of the causes of death in oncological patients. PE is the most serious complication of VTE, and patients with hepatocellular carcinoma (HCC) have an increased risk of developing PE. PE is a rare complication of HCC and may be a presenting symptom of HCC. The only or first symptom of PE can be syncope, which is a temporary loss of consciousness caused by a shortage of blood supply to the brain.^[3]^ It is rare that cases eventually diagnosed as HCC present with syncope as the initial PE symptom. In this article, we report 2 cases of syncope resulting from pulmonary thromboembolism that were later diagnosed with HCC, and review previous case reports of PE in HCC.

## 2 Case presentation

### 2.1 Case 1

**Figure 1:**
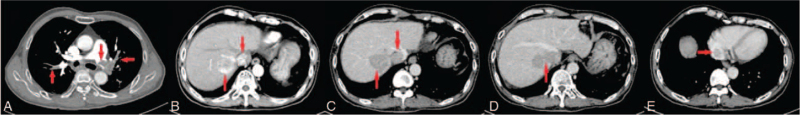
(A) CTPA showed embolization of left main pulmonary artery and branches of the both 2 pulmonary arteries; (B) A multiphasic contrast-enhanced abdominal CT showed HCC in the arterial phase; (C) HCC in the venous phase; (D) HCC invade IVC accompanied tumor emboli form in the venous phase; (E) HCC invade right atrium. CT = computerized tomography, CTPA = computed tomography pulmonary angiography, HCC = hepatocellular carcinoma, IVC = inferior vena cava.

A 65-year-old man was admitted for 2 episodes of syncope within 5 days. There was no chest pain, chest tightness, palpitation, or dyspnea before or after syncope. His previous history included 40 years of smoking, alcohol consumption, and chronic hepatitis B history of more than 20 years. His blood pressure was 120/70 mm Hg and there were no obvious positive signs on the physical examination on admission. Laboratory findings showed that D-dimer levels were greater than the upper limit of detection (>20 μg/mL, normal range, 0.00–0.50 μg/mL). An arterial blood gas analysis showed that arterial carbon dioxide tension was 33.0 mm Hg (normal range, 35.0–45.0 mm Hg) and arterial oxygen tension was 55.3 mm Hg (normal range, 80–100 mm Hg). Cardiac injury markers suggested troponin T 0.143 ng/mL (normal range, <0.014 ng/mL) and pro-BNP was 844 pg/mL (normal range, 0–125 pg/mL). Electrocardiogram readings showed sinus tachycardia and T-wave inversion in lead III. Head computerized tomography (CT) examination in the emergency department suggested subcortical lacunar cerebral infarction in the bilateral frontal lobe. Only suspicious density in the liver could be found on pulmonary CT and whole-abdomen multislice CT. Based on these results, we strongly suspected PE. Therefore, emergent computerized tomography pulmonary angiography (CTPA) was performed, and the results showed embolization of the left main pulmonary artery and branches of both pulmonary arteries (Fig. 1A), a suspected space-occupying lesion in the inferior vena cava (IVC) and right atrium. PE was diagnosed, alteplase was used for venous thrombolysis, and rivaroxaban was subsequently used for anti-coagulant therapy. No syncope recurred after thrombolysis. The oxygen partial pressure increased and the D-dimer level decreased. Color Doppler ultrasonography performed the following day revealed a slightly strong echo near the entrance of the right atrium and mild tricuspid regurgitation, but no abnormality was observed in the lower extremity vein. PE could not be explained by DVT of the lower extremity, and the cause was further investigated. Other laboratory tests showed that hepatitis B surface antigen >250 IU/mL (normal range 0–0.05 IU/mL), aspartate aminotransferase 57 U/L (normal range 15–40 U/L), gamma-glutamyl transpeptidase 72 U/L (normal range 10–60 U/L), albumin 27.8 g/L (normal range 40–55 g/L), and alpha-fetoprotein (AFP) 406 ng/mL (normal range 0–7.00 ng/mL). Combined with the patient's previous history of hepatitis B and abdominal CT indicated suspicious density in the liver, liver cancer was highly suspected. A multiphasic contrast-enhanced abdominal CT showed liver cirrhosis, ascites, and liver space-occupying lesions, indicating HCC (Fig. 1B,C). HCC invaded the IVC with the formation of tumor emboli (Fig. 1D), and the right atrium was also involved (Fig. 1E). The patient was recommended to the tumor hospital for the treatment of HCC after 1 week of hospitalization.

### 2.2 Case 2

**Figure 2:**
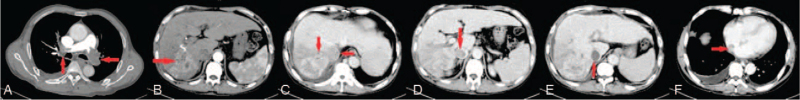
(A) CTPA performed and the result showed embolization of bilateral pulmonary trunk and their branches; (B) A multiphasic contrast-enhanced abdominal CT showed HCC in the arterial phase; (C) HCC accompanied by tumor thrombus in the right hepatic vein and IVC in the venous phase; (D) IVC tumor thrombus in the venous phase; (E) IVC throumbosis in the venous phase; (F) HCC invade right atrium. CT = computerized tomography, CTPA = computed tomography pulmonary angiography, HCC = hepatocellular carcinoma, IVC = inferior vena cava.

A 64-year-old man with chronic heavy smoking and drinking for many years presented with intermittent dyspnea for 2 weeks and aggravated with syncope for 1 day. He also had a history of varicose veins. On admission, his blood pressure was 94/63 mm Hg, his bilateral lungs were clear, there were no rales on auscultation, cardiac rhythm was regular, and there was no heart murmur in each auscultatory valve. Abdominal examination findings were unremarkable. Laboratory findings showed that D-dimer levels were 16.02 μg/mL (normal range, 0–0.5 μg/mL). An arterial blood gas analysis showed that arterial carbon dioxide tension was 32.1 mm Hg (normal range, 35.0–45.0 mm Hg) and arterial oxygen tension was 53.2 mm Hg (normal range, 80–100 mm Hg). Cardiac injury markers suggested troponin T 0.321 ng/mL (normal range, <0.014 ng/mL) and pro-BNP was 722 pg/mL (normal range, 0–125 pg/mL). Electrocardiogram showed sinus tachycardia with 100 beats per minute and T wave low and inverted in leads dominated by R waves. Based on these results, we strongly suspected PE. Therefore, emergent CTPA was performed, and the result showed embolization of the bilateral pulmonary trunk and branches (Fig. 2A). Immediately after confirmation of the PE diagnosis, rivaroxaban was administered. This was discontinued because hematuria appeared after 1 dose of rivaroxaban. The improvement was achieved and no syncope recurred after subcutaneous injection of low molecular weight heparin (LMWH), and the indicators related to PE were improved. Transthoracic echocardiography performed the following day revealed enlargement of the right atrium and right ventricle, widening of the left and right pulmonary arteries suspected emboli formation, and severe tricuspid regurgitation. However, a lower limb venous compression ultrasonography revealed no DVT, so the cause of PE was unknown. His liver function revealed that aspartate aminotransferase 115 U/L (normal range 15–40 U/L), alanine aminotransferase 103 U/L (normal range 15–40 U/L), alkaline phosphatase 229 U/L (normal range 45–125 U/L), and gamma-glutamyl transpeptidase 200 U/L (normal range 10–60 U/L), albumin 33.6 g/L (normal range 40–55 g/L), and liver changes on color Doppler ultrasonography at the outer hospital. Combined with a long history of heavy drinking, we considered the possibility of an underlying illness, such as cancer. Therefore, the male tumor markers and PE-related markers were screened. The results showed that AFP 1031 ng/mL (normal range 0–7.00 ng/mL). Color Doppler ultrasonography of the digestive system in our hospital indicated diffuse liver parenchyma lesions, slight echogenicity in liver suspected space-occupying lesions (approximately 51 mm × 35 mm) and splenomegaly. A multiphase liver protocol contrast-enhanced abdominal CT showed HCC (Fig. 2B) in the right posterior lobe of the liver accompanied by tumor thrombus in the right hepatic vein, tumor thrombus and thromboembolism IVC (Fig. 2C,D,E), and tumor thrombus in the right atrium (Fig. 2F). The patient was recommended to the tumor hospital for the treatment of HCC after 2 weeks’ hospitalization. Our case report was waived from the Ethical Board, based upon their policy to review all intervention and observational study except for a case report. These 2 patients provided informed consent for the publication of their clinical data. The presented data were anonymized, and the risk of identification was minimal.

### 2.3 Literature review

Our literature search for related cases identified 27 HCC patients with PE in 27 articles^[4–30]^ from 1962 to 2020 in the PubMed database (Table 1). These patients were aged between 16 and 83 years (mean age: 54 years), and 85% of the patients were men. Only 22.2% of patients had a history of HCC, and the other 77.8% patients did not have a history of HCC. Except for 1 case of mixed HCC combined with intrahepatic cholangiocarcinoma (ICC), all other pathologic types were HCC. Regarding the underlying disease, 48.1% had hepatitis B or C, 14.8% had liver cirrhosis associated with hepatitis or not, 14.8% had alcohol intake, 3.7% had hepatitis C and excessive alcohol consumption, 7.4% had no reason, and the reason for the other 11.1% were not available (NA). Regarding the initial presenting symptoms of HCC with PE, up to 44.4% had PE as the first manifestation of HCC, HCC (25.9%), HCC plus PE (11.1%), liver cirrhosis, progressive ascites upper gastrointestinal bleeding, right heart failure, acute myocardial infarction, and NA in 3.7% of patients. Of the HCC cases, 48.1% were diagnosed with autopsy or combined with other methods, 16.7% with biopsy or combined with other imaging methods, 37.5% with imaging methods, and 3.7% with NA. A total of 29.6% of HCC patients who received treatment included 22.2% hepatectomy or combined with other methods, transcatheter arterial chemoembolization, and sorafenib (3.7%). 48.1% of HCC patients did not receive treatment because 18.5% were found postmortem, 18.5% received palliative care and death occurred before treatment in 11.1%, and 22.2% were NA. 37% tumor or tumor thrombus involved IVC, 3.7% involved the hepatic veins, 29.6% involved both the hepatic veins or IVC, neither the hepatic vein nor the IVC was involved in 14.8%, and 14.8% were NA. Approximately 40.7% of tumors or tumor thrombus involved right atrium, 3.7% involved the right ventricle, neither the right atrium nor ventricle was involved in 40.7%, and 14.8% were NA. Only 18.5% had pulmonary hypertension, 63% had no pulmonary hypertension, and 18.5% were NA. For the first symptom of PE, most (72.7%) showed dyspnea or shortness of breath or combined with other symptoms, 7.4% showed cardiopulmonary arrest, high fever with recurrent pneumonia, hemoptysis, and asymptomatic PE found postmortem (3.7%), and 11.1% were NA. Of these, 48.1% were diagnosed with autopsy or combined with other methods, 48.1% with imaging methods, and 3.7% were NA. Regarding the properties of pulmonary emboli, 63% were tumoral thrombi, 7.4% were tumoral and mixed thrombi, 3.7% were non-traumatic fat emboli, and the remaining 25.9% were NA. Regarding the PE treatment, 40.7% of patients received no treatment because PE was found postmortem, 18.5% received anti-coagulation therapy, 7.4% received thrombolysis and subsequent anti-coagulation, 7.4% received surgery combined with anti-coagulation, 3.7% received aspiration with a catheter, and 22.2% were NA. Only 3.7% of the PE patients had DVT, while the others without DVT or were NA. Regarding outcomes, only 22.2% patients had an improved prognosis, 63% of patients died, of which 14.8% died of PE, 11.1% may be poor because palliative care was adopted, and 3.7% were NA.

**Table 1:** Reported hepatocellular carcinoma patients with pulmonary embolism.

Case	Author/year	Age	Sex	History of HCC	HCC initial symptom	Underlying disease	Diagnostic tool of HCC	Types of HCC
1	Present case 1	65	Male	No	PE	Chronic hepatitis B	Contrast-enhanced abdominal CT	HCC
2	Present case 2	64	Male	No	PE	Extensive drinking	Contrast-enhanced abdominal CT	HCC
3	Filippos-Paschalis Rorris/2020	53	Male	No	PE	HCV infection	Staging CT scan of abdomen	HCC
4	Kensuke Yamamura/2020	83	Female	No	HC	NA	Contrast-enhanced CT, biopsy	HCC
5	Luís C Lourenço/2017	47	Male	No	HC	Chronic hepatitis C	Contrast-enhanced abdominal CT	HCC
6	Mai Sakashita/2017	81	Male	Yes	NA	Alcoholic cirrhosis	Autopsy	HCC
7	Nobuyuki Yamashita/2015	60	Female	Yes	PE	NA	Autopsy	HCC
8	Toshimasa Clark/2014	65	Male	No	PE and HC	Chronic hepatitis C	Contrast-enhanced abdominal CT and autopsy	HCC
9	Cheng-Hsien Wu/2013	68	Male	Yes	PE	Chronic hepatitis B and C	CTPA	HCC
10	Sumeet K Asrani/2012	21	Male	No	HC and PE	None	Abdominal CT scan and liver biopsy	HCC
11	Hsin-Kai Huang/2011	64	Male	No	PE	Reactive anti-HCV antibody	Histological examination and contrast-enhanced CT	HCC
12	Vikrant Nayar/2010	59	Female	No	Right heart failure	Hepatitis C and excessive alcohol consumption	Ultrasound and contrast-enhanced CT	HCC
13	Carlos Gilberto Canelo Aybar/2008	16	Male	No	PE	NA	Autopsy	HCC
14	Hsuan-Hwai Lin/2007	57	Male	No	PE	Chronic hepatitis B	Abdominal ultrasonography, abdominal CT and MRI	HCC
15	Mitsuru Nakanishi/2006	27	Male	Yes	Upper gastrointestinal bleeding	NA	CT	HCC
16	Jörg Jäkel/2006	48	Male	No	Liver cirrhosis, progressive ascites	Alcohol abuse and subsequent liver cirrhosis	Autopsy	HCC
17	Chun-Lin Chi/2005	34	Male	Yes	PE and HC	NA	Abdominal ultrasonography and chest CT	HCC
18	Elod Papp/2005	63	Male	No	HC	Hepatitis B or C viral infection	Abdominal ultrasound, CT scan, fine needle biopsy and autopsy	HCC
19	O Diaz Castro/2004	71	Male	No	AMI	Chronic hepatitis C	Autopsy, pathological findings	HCC
20	Alfonso Gutiérrez-Macías/2002	41	Male	No	PE	Heavy alcohol	Postmortem examination	HCC
21	K Wilson/2001	65	Male	No	PE	None	Abdominal contrast-enhanced CT and angiography of the IVC	HCC
22	J Koskinas /2000	30	Female	No	PE	HBsAg-positive	Autopsy	HCC-ICC
23	G S Chan/2000	52	Male	No	HC	Chronic hepatitis B	CT and autopsy	HCC
24	T Mularek-Kubzdela/1996	49	Male	No	PE	Hepatitis B	Abdominal sonography and CT	HCC
25	N Masaki/1994	48	Male	Yes	HC	HBV carrier	Ultrasonography and angiography	HCC
26	J Murayama/1992	61	Male	No	HC	Liver cirrhosis associated with HB viral chronic hepatitis	Autopsy	HCC
27	Kolarski V/1990	73	Male	No	HC	Macronodular liver cirrhosis	NA	HCC
28	J U Brisbane/1980	63	Male	No	PE	Regular alcohol	Liver scan and biopsy	HCC
29	P B STOREY/1962	58	Male	No	PE	Posthepatitic cirrhosis of liver with hepatitis	Autopsy	HCC

Case	HCC treatment	Tumor thrombus involves the hepatic veins or IVC	Tumor thrombus involves the hepatic veins or IVC	Pulmonary hypertension	First symptom of PE
1	No, cancer hospital is recommended	IVC	Right atrium	No	Syncope
2	No, cancer hospital is recommended	IVC	None	Yes	Dyspnea and syncope
3	Urgent operation	IVC	Right atrium	No	NA
4	Hepatectomy, liver resection, chemoembolization	Hepatic veins	None	No	NA
5	No, best supportive care	IVC	Right atrium	No	Shortness of breath
6	No, death occurred before treatment	None	None	No	CPA
7	Sorafenib	NA	NA	NA	Dyspnea
8	Comfort care measures	Both	Right atrium	No	Increased dyspnea on exertion
9	A segmental hepatectomy and transarterial embolization	IVC	None	No	Dyspnea, tightness in the chest and episodes of dizziness
10	No, hospice care with symptomatic palliation	IVC	Right atrium	No	New onset of shortness of breath
11	NA	IVC	Right atrium	Yes	Progressive dyspnea and chest pain
12	Palliative care	Both	Right atrium	No	Breathlessness
13	NA	NA	NA	NA	Cardiac insufficiency and cor pulmonare
14	Right hepatectomy	Both	Right atrium	No	Severe substernal chest pain then dyspnea and hemoptysis
15	NA	Both	None	No	Sudden chest pain and dyspnea
16	No, HC was found postmortem	None	Right ventricle	No	Asymptomatic, PE was found postmortem
17	NA	IVC	None	Yes	Shortness of breath
18	Combined liver and heart surgery	IVC	Right atrium	No	High fever, recurrent pneumonias
19	No, death occurred before treatment	Both	Right atrium	No	A sudden pleuritic chest pain, with severe dyspnea
20	No, HC was found postmortem	None	None	No	Shortness of breath
21	Excision of IVC mass and partial hepatic venous component	Both	Right atrium	Yes	Recurrent episodes of dyspnea
22	No, HC was found postmortem	NA	None	Yes	Progressive shortness of breath
23	No, death Occurred before treatment	Both	None	no	Sudden tonic-clonic convulsion shortly followed by CPA
24	NA	IVC	Right atrium	NA	Shortness of breath
25	TAE	IVC	None	No	Exertional dyspnea
26	No, HC was found postmortem	NA	NA	NA	Hemoptysis
27	NA	IVC	NA	NA	NA
28	Multifocal hepatocarcinoma, was not treated with chemotherapy	None	None	No	Shortness of breath
29	No, HC was found postmortem	Both	None	Yes	Right-sided chest pain and shortness of breath

Case	Diagnosic tool of PE	Properties of emboli	PE treatment	DVT	Prognosis	Note	Ref.
1	CTPA	Thrombus	rt-PA, then rivaroxaban	No	Improve		
2	CTPA	Thrombus	LMWH when Hematuria occurred after one dose of rivaroxaban	No	Improve		
3	CTPA	NA	NA	NA	Improve	HCC in the adrenal gland	^[4]^
4	Coronal equilibrium-phase CT image	NA	Edoxaban	Yes	Improve		^[5]^
5	Chest CT	NA	NA	NA	Discharged with best supportive care		^[6]^
6	Autopsy	Non-traumatic FES	No, PE was found postmortem	NA	Died of PE		^[7]^
7	Autopsy	Tumoral thrombi	No, PE was found postmortem	NA	Died of respiratory failure	English abstracts only	^[8]^
8	Autopsy	Tumor emboli	No, PE was found postmortem	NA	Died of respiratory failure and asystole secondary to lactic acidosis		^[9]^
9	CTPA	Tumor embolus	Intravenous heparin and surgical removal of the embolus	no	Died with cardiac arrhythmias		^[10]^
10	Chest CT	Both tumor thrombus and bland thrombus	NA	NA	Discharged to hospice care with symptomatic palliation		^[11]^
11	Contrast-enhanced CT	Tumor emboli	NA	NA	Improve		^[12]^
12	NA	NA	NA	NA	Discharged home for palliative care		^[13]^
13	Autopsy	Tumoral thrombi	No, PE was found postmortem	NA	Died	English abstracts only	^[14]^
14	Ventilation–perfusion lung scan	NA	Anticoagulant	NA	Died of multiple organ failure		^[15]^
15	Emergency CT scan and pulmonary angiography	Tumor emboli	The emboli was removed by aspiration with a catheter	NA	Improve		^[16]^
16	Autopsy	Tumor emboli	No, PE was found postmortem	NA	Developed an intractable hepato-renal syndrome and died		^[17]^
17	Spiral CT scan of chest and Ventilation-perfusion scan	Tumor emboli	LMWH	NA	Improve		^[18]^
18	Autopsy	Tumor emboli	No, PE was found postmortem	NA	Acute cor pulmonale occurred during tumor removal from the right atrium and the patient expired		^[19]^
19	Pulmonary arteriography	Tumor emboli	Intra-arterial thrombolysis with urokinase	NA	Died 4 hours after thrombolysis	The same tumorous cells in the distal LCX and LAD	^[20]^
20	Ventilation-perfusion lung scan, helical CT scan and Postmortem examination	Tumor emboli	rt-PA and intravenous unfractionated heparin	NA	Died, death reason NA		^[21]^
21	Ventilation-perfusion lung scan	Tumor emboli	Heparin, followed by warfarin	NA	Recovered uneventfully	No HCC in the liver	^[22]^
22	Autopsy	NA	No, PE was found postmortem	NA	Died of PE	English abstracts only	^[23]^
23	Autopsy	Tumor emboli	No, PE was found postmortem	No	Died of massive pulmonary tumor embolism		^[24]^
24	Pulmonary perfusion scintigraphy	NA	NA	NA	NA	English abstracts only	^[25]^
25	CT scan and MRI	Tumoral thrombi	Tumor thrombus was removed using Open-heart surgery	NA	Died, death reason NA		^[26]^
26	Autopsy	Thromboembolism	No, PE was found postmortem	NA	Died of PE	English abstracts only	^[27]^
27	Autopsy	Tumor fragments	No, PE was found postmortem	NA	Died	English abstracts only	^[28]^
28	Autopsy	Tumoral, platelet-fibrin, and mixed thrombi	No, PE was found postmortem	NA	Died		^[29]^
29	Chinical diagnosis and autopsy	Tumoral thrombi	Warfarin, discontinuation because of excessive response to warfarin	NA	Died		^[30]^

CPA = cardiopulmonary arrest, CT = computerized tomography, CTPA = computed tomography pulmonary angiogram, DVT = deep venous thrombosis, FES = fat embolism syndrome, HBV = chronic hepatitis B, HCC = hepatocellular carcinoma, HCV = chronic hepatitis C, ICC = intrahepatic cholangiocarcinoma, IVC = inferior vena cava, LAD = left anterior descending coronary artery, LCX = left circumflex coronary artery, LMWH = low molecular weight heparin, MRI = Magnetic resonance imaging, NA = not available, PE = pulmonary embolism, TACE = transcatheter arterial chemoembolization, TAE = transcatheter arterial embolization.

## 3 Discussion

Primary liver cancer is the fourth most common malignant tumor in China and the third most lethal cause of cancer, and a serious threat to the life and health of Chinese people.^[31,32]^ The pathological type of primary liver cancer includes HCC, accounting for 85% to 90%,^[33]^ and a few are ICC and mixed with HCC-ICC. People at high risk for HCC are mainly those with HBV and/or HCV infection, chronic alcohol abuse (alcoholic liver disease), non-alcoholic steatohepatitis, consumption of aflatoxin-contaminated foods, cirrhosis of the liver from a variety of causes, and a family history of liver cancer. Patients with cancer have a 4 to 7-fold higher risk of developing VTE, which includes DVT and PE.^[34]^ Most PE occurs in patients with malignant tumors of the HCC, breast, renal, and gastric tumors and PE has been reported in approximately 8% of patients with HCC.^[35]^

In our review, we found that the majority of HCCs combined with PE occurred in middle-aged and older men. Most of the patients were associated with viral hepatitis B or C, followed by liver cirrhosis and alcohol intake, which is consistent with the epidemiology of our country. Very few people have a clear history of primary liver cancer prior to onset and PE was the primary manifestation in nearly half of the patients with HCC combined with PE. More than half of the patients with HCC were diagnosed by biopsy or autopsy. The diagnosis of HCC in our cases was not pathologically confirmed but could be supported by elevated AFP and enhanced abdominal CT. Both cases showed uneven and significant arterial-phase hyperenhancement which is the major feature of HCC according to the Liver Imaging Reporting and Data System.^[36]^ Significantly reduced tumor enhancement in the portal phase was also observed. Such fast-in-and-out enhancement is a typical imaging feature of HCC.^[37]^

At the initial diagnosis of HCC, our patient had PE which was the sole presentation without any of the typical manifestations of HCC. PE has a variety of symptoms and the most frequent symptoms are dyspnea or shortness of breath, which is consistent with the reviewed literature. Although syncope can be the only or initial symptom of PE, there were no cases of syncope in patients with HCC combined with PE. Syncope as the initial presentation of PE in HCC patients is particularly rare, but we reported 2 cases that were different from those reported in the literature. The frequency of syncope in patients with high-risk PE was 29.9%.^[38]^ Arterial hypotension and reduced cerebral blood flow caused by a significant decrease in cardiac output when thrombosis of more than half of the lung arterial system causes activation of the vasovagal reflex, arrhythmias, and conduction disturbances caused by an overload of the right ventricle are the possible mechanisms off syncope in patients with PE.^[39]^ These 2 patients in our study did not describe liver discomfort, but were admitted to our department with syncope. Based on D-dimer measurements, blood gas analysis, and CTPA, we clearly diagnosed PE. Because of the absence of long-term bed rest and DVT in the lower limbs, PE could not be explained by common causes. Thus, we further examined the underlying disease that causes PE and found HCC, IVC, and even right atrium tumor thrombus.

PE in patients with HCC may result from thromboembolism or embolism. The source of pulmonary thromboembolism in patients mainly originates from the deep veins in the legs or pelvis. Liver cancer is strongly associated with VTE^[40]^ and hypercoagulability in malignancy is a well-known cause. Malignant cells can activate blood coagulation by producing procoagulant activities, by releasing proinflammatory and proangiogenic cytokines, or by interacting directly with host vascular and blood cells,^[41]^ thereby promoting thrombosis formation. HCC is commonly associated with hepatic vein and IVC permeation, and subsequent secondary thrombosis may be another infrequent source of pulmonary thromboembolism, while tumor extension to the hepatic vein or IVC. Another less common source of pulmonary thromboembolism is secondary thrombosis caused by local pulmonary vascular endothelial damage. Most tumor cells are destroyed in the lung; however, some of them may survive and lodge in small vessels, producing a variable degree of endothelial injury that contributes to the formation of PE.^[42,43]^ However, it is more easily explained in HCC patients with PE because tumor embolism is usually related to invasion of veins by the tumor and contains a variable number of malignant cells. PE could be caused by metastatic HCC to the hepatic vein or IVC, even to the right atrium, as well as small metastatic tumor emboli. One study showed that significant pulmonary tumor embolism occurred in 3 (43%) of the 7 HCCs with evidence of major hepatic vein and IVC invasion.^[44]^

Although PE mainly tumor embolism is seen in up to 26% of autopsies, it is less frequently identified before death.^[45]^ Macroscopic and microscopic PE have been reported in HCC.^[20–22,24,46]^ Macroembolism could be represented as a large tumor embolus or thromboembolism secondary to the tumor that detaches from the IVC, passes into the right heart and finally enters to the pulmonary trunk. Microvascular embolization might result from the detachment of a small thrombus or tumor emboli of the deep vein, hepatic vein, or IVC. Larger fragments of the tumor or thrombus entered the right ventricle, which was then mechanically disrupted by the heart action, leading to pulmonary massive microembolism. Tumor cells enter the systemic circulation by invading vessels or through the tumor's own microvasculature, which produces a variable degree of endothelial injury that contributes to the formation of thrombi. Most case reports on tumor microembolism show a vascular tissue reaction ^[47–50]^ characterized by intimal proliferation and fibrosis.^[51]^

Tumor-associated thromboembolism or tumor embolization can range from massive saddle emboli complicated by cardiovascular collapse and death to asymptomatic microemboli detected only at autopsy. It may occur at any time in the natural history of neoplastic diseases and, in exceptional cases such as our report, may be the first manifestation of an occult carcinoma.^[23,48,52]^ It was luck for these 2 patients in our cases because syncope – the unique manifestation of massive PE – enabled these 2 patients to seek treatment early although these patients did not present with HCC. However, the possibility of PE should be considered in HCC patients presenting with acute dyspnea and a rapidly deteriorating clinical course, especially if there is evidence of venous permeation by the tumor.

Tumor macroembolism is clinically undistinguishable from massive pulmonary thromboembolism, and the correct diagnosis is mostly achieved through autopsy. Although there was no venous thrombosis of DVT in the lower extremity and pelvic vein, we believe that the nature of PE emboli in these 2 cases we reported was thromboemboli rather than tumor emboli, for several reasons. High levels of D-dimer and fibrin degradation products indicate hypercoagulability and secondary hyperfibrinolysis in vivo which could occur in thromboembolic disease. CTPA indicated that the emboli in the pulmonary artery were uniformly low-density and not enhanced. In addition to tumor tissue, thrombus could also be found in the IVC on contrast-enhanced CT of the abdomen, which could explain the source of PE. After anti-coagulant or thrombolytic therapy, the symptoms related to PE improved, D-dimer and fibrin degradation decreased and oxygen partial pressure increased, which also indirectly supported the thrombotic nature of the emboli. PE in our cases is therefore thought to have been caused by tumor-related hypercoagulability and tumor secondary thrombosis.

Early clinical diagnosis of PE in dyspneic patients with HCC has become increasingly important and may offer a better chance of survival. According to the proposed diagnostic algorithm in the updated guidelines of the European Society of Cardiology, an emergency CT scan is the first-line imaging tool for patients with suspected high-risk PE.^[53]^ Compared with conventional CT, the sensitivity of multirow detector spiral CT for acute PE increased from approximately 70% to more than 90%.^[54,55]^ The major advantages of spiral CT are that the thrombus can be directly visualized and alternative diagnosis can be established on lung parenchymal images that are not evident on chest radiography. However, for patients with a definite diagnosis of PE, tumor markers may be a preferred method of screening to identify the underlying neoplastic disease if PE cannot be explained by the common cause. When right atrial space-occupying lesions were found in CTPA, we should consider that it may be associated with IVC lesions. Identification of IVC thrombus by abdominal ultrasonography is crucial because the sentinel sign would alert caregivers to arrange further surveys for suspected pulmonary tumor embolism or thromboembolism. Although relatively rare, tumor embolization or tumor-associated thrombosis should always be considered in cases of PE, even in the absence of a history of malignancy. Our cases remind physicians to focus on space-occupying lesions of the right atrium when PE is confirmed by CTPA. Whether the right atrial space-occupying lesion is connected to the IVC or whether the space-occupying lesion of the right atrium is a continuation of abdominal neoplastic disease should be further explored. The cases also remind us that tumor markers may be a preferred method of screening to identify the underlying neoplastic disease if PE cannot be explained by the common cause.

Given that the pulmonary embolus can be of tumor origin, tumor embolus or tumor-associated thrombosis, embolectomy or even lobectomy can be a useful therapeutic modality,^[56]^ even in cases with extension of HCC to the right side of the heart.^[26]^ However, the surgical approach to IVC and intra-atrial masses is difficult, and it is impossible to completely remove the tumor thrombi from the peripheral pulmonary arteries. Unless new extension of the tumor thrombus is prevented by successive treatments for the primary liver tumor, the effects of open-heart surgery will be transient and limited. The outcome of the operation is unpredictable, most patients usually die within a short period because of PE, heart failure, or cancer progression.^[19]^ For those who survived, the subsequent course was determined by whether growth of the residual tumor could be controlled by successive multidisciplinary treatments. We found that the 1-year survival rates of patients with or without surgery were 40% and 0% ^[15]^ and resection can provide relatively good long-term survival but not more than 2 years.^[15,45]^ Therefore, hepatic resection with removal of tumor thrombi should be considered to prolong a patient's life span. Other approaches should also be considered, in addition to hepatic resection with removal of the tumor thrombus. Aspiration with a catheter resulted in a reduction in the size of the emboli, and the patient showed symptomatic improvement.^[16]^ Stent implantation into the IVC may be helpful in preventing tumor emboli from floating into and blocking the major pulmonary vasculature. Various adjuvant systemic treatment modalities including chemotherapy, immunotherapy, and hormone therapy, are still of limited value in HCC.^[57–59]^ For PE caused by thromboembolism in HCC, thrombolysis and anti-coagulation are the 2 main treatment methods. It is important to exclude non-thrombotic PEs before initiating thrombolytic therapy. Thrombolytic therapy is the treatment of choice for patients with thromboembolic PE presenting with shock or arterial hypotension.^[53]^ LMWH is a standard treatment regimen for cancer-associated thrombosis^[60,61]^; however, a recently unique network meta-analysis of randomized control trials demonstrated that the effectiveness and safety of new oral anti-coagulants were non-inferior to vitamin K antagonists, and possibly comparable with LMWH for the treatment of cancer associated thrombosis.^[62]^ The cases we reported improved after aggressive thrombotic or anti-coagulant therapy when pulmonary thromboembolism was confirmed. Unfortunately, we could not treat HCC, IVC, and right atrial tumor thrombus due to the lack of oncology departments in our hospital.

### 3.1 Limitations

This study had several limitations. HCC with pulmonary thromboembolism is a clinical diagnosis that includes laboratory tests and imaging tests, but is not pathologically confirmed. In addition, the treatment of HCC is unknown because there is no oncology department in our hospital, and the prognosis of PE has not been observed. Nevertheless, we reported 2 cases of HCC combined with PE before autopsy.

## 4 Conclusion

In conclusion, we described 2 rare cases of HCC complicated by PE who first presented with syncope. After a series of examinations, HCC complicated with tumor thrombi in the IVC and right atrium was observed. It is important for clinicians to be aware that occult carcinoma might be a reason for patients with PE presenting with syncope if PE cannot be explained by common causes such as in the cases of our patient, and HCC should be highly suspected when IVC and right atrial mass was found on imaging tests.

## Acknowledgment

We thank all participants for their support and participation.

## Author contributions

**Conceptualization:** Dayong Deng, Haidi Wu.

**Data curation:** Zikai Song.

**Formal analysis:** Huafang Wei.

**Methodology:** Yang Yu.

**Software:** Chongyin Zhang.

**Writing – original draft:** Dayong Deng, Lei Yang.

**Writing – review & editing:** Haidi Wu.

## References

[1] Serrano PE Parpia S Linkins LA. Venous thromboembolic events following major pelvic and abdominal surgeries for cancer: a prospective cohort study. Ann Surg Oncol 2018;25:3214–21. 30051364 10.1245/s10434-018-6671-7

[2] Chew HK Wun T Harvey D Zhou H White RH. Incidence of venous thromboembolism and its effect on survival among patients with common cancers. Arch Intern Med 2006;166:458–64. 16505267 10.1001/archinte.166.4.458

[3] Task Force for the D, Management of S, European Society of C, et al. Guidelines for the diagnosis and management of syncope (version 2009). Eur Heart J 2009;30: 2631–2671. 10.1093/eurheartj/ehp298 PMC3295536 19713422

[4] Rorris FP Tyrovolas K Theodosis A. Ectopic hepatocellular carcinoma in the adrenal gland with inferior vena cava thrombosis and right atrial extension. J Card Surg 2020;35:1380–2. 32353896 10.1111/jocs.14594

[5] Yamamura K Beppu T Kinoshita K. Hepatocellular carcinoma with extensive cancer-associated thrombosis successfully treated with liver resection and direct oral anticoagulant: a case report. Anticancer Res 2020;40:6465–71. 33109585 10.21873/anticanres.14668

[6] Lourenco LC Horta DV Alberto SF Reis J. Hepatocellular carcinoma presenting with Budd-Chiari syndrome, right atrial thrombus and pulmonary emboli. Rev Esp Enferm Dig 2017;109:296–7. 28372458

[7] Sakashita M Sakashita S Sakata A. An autopsy case of non-traumatic fat embolism syndrome. Pathol Int 2017;67:477–82. 28667706 10.1111/pin.12556

[8] Yamashita N Tanimoto H Yamamoto H Nishiura S Nomura H. Hypoxemia due to pulmonary tumor microembolisms from a hepatocellular carcinoma: a case report. Nihon Shokakibyo Gakkai Zasshi 2015;112:1060–6. 26050730 10.11405/nisshoshi.112.1060

[9] Clark T Maximin S Shriki J Bhargava P. Tumoral pulmonary emboli from angioinvasive hepatocellular carcinoma. Curr Probl Diagn Radiol 2014;43:227–31. 24948215 10.1067/j.cpradiol.2014.04.006

[10] Wu CH Wong YC Wu MY Nan YY Lin WL. Education and imaging: hepatobiliary and pancreatic: fatal tumor embolism caused by hepatocellular carcinoma. J Gastroenterol Hepatol 2013;28:380. 23339392 10.1111/jgh.12048

[11] Asrani SK LaRusso NF. Fibrolamellar hepatocellular carcinoma presenting with Budd-Chiari syndrome, right atrial thrombus, and pulmonary emboli. Hepatology 2012;55:977–8. 22105537 10.1002/hep.24811

[12] Huang HK Huang CH Wu CC Chen WJ. An unusual cause of acute pulmonary embolism. Int J Cardiol 2011;149:e88–9. 20591513 10.1016/j.ijcard.2010.05.058

[13] Nayar V Singh B Pugh PJ. Hepatocellular carcinoma presenting as right heart failure. Eur Heart J 2010;31:1957. 20494904 10.1093/eurheartj/ehq147

[14] Canelo Aybar CG Cuadra Urteaga JL Fujii F Romaní FR Matute FAA Rubio ROV. Tumoral microembolism and cor-pulmonar as manifestation of hepatocelular carcinoma. Rev Gastroenterol Peru 2008;28:70–3. 18418460

[15] Lin HH Hsieh CB Chu HC Chang WK Chao YC. Acute pulmonary embolism as the first manifestation of hepatocellular carcinoma complicated with tumor thrombi in the inferior vena cava: surgery or not? Dig Dis Sci 2007;52:1554–7. 17357843 10.1007/s10620-006-9129-x

[16] Nakanishi M Hige S Chuma M Asaka M. Pulmonary tumor embolism in a case of hepatocellular carcinoma. J Gastroenterol 2006;41:808–9. 16988773 10.1007/s00535-006-1850-x

[17] Jakel J Ramaswamy A Kohler U Barth PJ. Massive pulmonary tumor microembolism from a hepatocellular carcinoma. Pathol Res Pract 2006;202:395–9. 16488087 10.1016/j.prp.2006.01.005

[18] Chi CL Liu KL Yuan A Lien WC Chen WJ Wang HP. Pulmonary tumor embolism--diagnosis in the ED. Am J Emerg Med 2005;23:808–10. 16182993 10.1016/j.ajem.2005.03.010

[19] Papp E Keszthelyi Z Kalmar NK. Pulmonary embolization as primary manifestation of hepatocellular carcinoma with intracardiac penetration: a case report. World J Gastroenterol 2005;11:2357–9. 15818754 10.3748/wjg.v11.i15.2357 PMC4305827

[20] Diaz Castro O Bueno H Nebreda LA. Acute myocardial infarction caused by paradoxical tumorous embolism as a manifestation of hepatocarcinoma. Heart 2004;90:e29. 15084577 10.1136/hrt.2004.033480 PMC1768223

[21] Gutierrez-Macias A Barandiaran KE Ercoreca FJ Zárate MMO. Acute cor pulmonale due to microscopic tumour embolism as the first manifestation of hepatocellular carcinoma. Eur J Gastroenterol Hepatol 2002;14:775–7. 12169988 10.1097/00042737-200207000-00011

[22] Wilson K Guardino J Shapira O. Pulmonary tumor embolism as a presenting feature of cavoatrial hepatocellular carcinoma. Chest 2001;119:657–8. 11171756 10.1378/chest.119.2.657

[23] Koskinas J Betrosian A Kafiri G Tsolakidis G Garaziotou V Hadziyannis S. Combined hepatocellular-cholangiocarcinoma presented with massive pulmonary embolism. Hepatogastroenterology 2000;47:1125–8. 11020895

[24] Chan GS Ng WK Ng IO Dickens P. Sudden death from massive pulmonary tumor embolism due to hepatocellular carcinoma. Forensic Sci Int 2000;108:215–21. 10737468 10.1016/s0379-0738(99)00212-1

[25] Mularek-Kubzdela T Stachowiak W Grajek S. A case of primary hepatocellular carcinoma with tumor thrombus in the right atrium and massive pulmonary embolism. Pol Arch Med Wewn 1996;95:245–9. 8755855

[26] Masaki N Hayashi S Maruyama T. Marked clinical improvement in patients with hepatocellular carcinoma by surgical removal of extended tumor mass in right atrium and pulmonary arteries. Cancer Chemother Pharmacol 1994;33 Suppl:S7–11. 8137487 10.1007/BF00686660

[27] Murayama J Naitoh T Doi M. A case of small liver cancer presenting as a huge mediastinal mass. Nihon Kyobu Shikkan Gakkai Zasshi 1992;30:708–13. 1328737

[28] Kolarski V Dimitrova B Chemishanski G. Pulmonary tumor embolism in primary cancer of the liver. Vutr Boles 1990;29:84–8. 2173285

[29] Brisbane JU Howell DA Bonkowsky HL. Pulmonary hypertension as a presentation of hepatocarcinoma. Report of a case and brief review of the literature. Am J Med 1980;68:466–9. 6244734 10.1016/0002-9343(80)90123-0

[30] Storey PB Goldstein W. Pulmonary embolization from primary hepatic carcinoma. Arch Intern Med 1962;110:262–9. 13917519 10.1001/archinte.1962.03620200122021

[31] Torre LA Bray F Siegel RL Ferlay J Lortet-Tieulent J Jemal A. Global cancer statistics, 2012. CA Cancer J Clin 2015;65:87–108. 25651787 10.3322/caac.21262

[32] Chen W Zheng R Baade PD. Cancer statistics in China, 2015. CA Cancer J Clin 2016;66:115–32. 26808342 10.3322/caac.21338

[33] Guidelines CW. Chinese Socity of Clinical Oncology (CSCO) Guidelines for the Diagnosis and Treatment of Hepatocellular Carcinoma 2018. V1. Beijing: People's Medical Publishing House; 2018.

[34] Voigtlaender M Langer F. Management of cancer-associated venous thromboembolism - a case-based practical approach. Vasa 2018;47:77–89. 29325495 10.1024/0301-1526/a000684

[35] Hulman G. The pathogenesis of fat embolism. J Pathol 1995;176:03–9. 10.1002/path.1711760103 7616354

[36] Elsayes KM Kielar AZ Agrons MM. Liver Imaging Reporting and Data System: an expert consensus statement. J Hepatocell Carcinoma 2017;4:29–39. 28255543 10.2147/JHC.S125396 PMC5322844

[37] Forner A Vilana R Ayuso C. Diagnosis of hepatic nodules 20 mm or smaller in cirrhosis: prospective validation of the noninvasive diagnostic criteria for hepatocellular carcinoma. Hepatology 2008;47:97–104. 18069697 10.1002/hep.21966

[38] Duplyakov D Kurakina E Pavlova T. Value of syncope in patients with high-to-intermediate risk pulmonary artery embolism. Eur Heart J Acute Cardiovasc Care 2015;4:353–8. 24619817 10.1177/2048872614527837

[39] Morrone D Morrone V. Acute pulmonary embolism: focus on the clinical picture. Korean Circ J 2018;48:365–81. 29737640 10.4070/kcj.2017.0314 PMC5940642

[40] Sorensen HT Mellemkjaer L Steffensen FH. The risk of a diagnosis of cancer after primary deep venous thrombosis or pulmonary embolism. N Engl J Med 1998;338:1169–73. 9554856 10.1056/NEJM199804233381701

[41] Prandoni P Falanga A Piccioli A. Cancer and venous thromboembolism. Lancet Oncol 2005;6:401–10. 15925818 10.1016/S1470-2045(05)70207-2

[42] Bassiri AG Haghighi B Doyle RL Berry GJ Rizk NW. Pulmonary tumor embolism. Am J Respir Crit Care Med 1997;155:2089–95. 9196119 10.1164/ajrccm.155.6.9196119

[43] Soares FA Pinto AP Landell GA de Oliveira JA. Pulmonary tumor embolism to arterial vessels and carcinomatous lymphangitis. A comparative clinicopathological study. Arch Pathol Lab Med 1993;117:827–31. 8343048

[44] Ryder SD. British Society of G. Guidelines for the diagnosis and treatment of hepatocellular carcinoma (HCC) in adults. Gut 2003;52 Suppl 3:iii1–8. 12692148 10.1136/gut.52.suppl_3.iii1 PMC1867754

[45] Fujisaki M Kurihara E Kikuchi K. Hepatocellular carcinoma with tumor thrombus extending into the right atrium: report of a successful resection with the use of cardiopulmonary bypass. Surgery 1991;109:214–9. 1846985

[46] Chai L Ong KC Ng SB. A case of pulmonary tumour embolism mimicking miliary tuberculosis. Respirology 2000;5:297–9. 11022995 10.1046/j.1440-1843.2000.00262.x

[47] Chinen K Kazumoto T Ohkura Y Matsubara O Tsuchiya E. Pulmonary tumor thrombotic microangiopathy caused by a gastric carcinoma expressing vascular endothelial growth factor and tissue factor. Pathol Int 2005;55:27–31. 15660700 10.1111/j.1440-1827.2005.01783.x

[48] Montero A Vidaller A Mitjavila F Chivite D Pujol R. Microscopic pulmonary tumoral embolism and subacute cor pulmonale as the first clinical signs of cancer. Acta Oncol 1999;38:1116–8. 10665773 10.1080/028418699432464

[49] Steiner S Plehn G Reinecke P. Disseminated microvascular pulmonary tumor cell embolism: a rare cause of fulminant pulmonary hypertension. Onkologie 2004;27:566–8. 15591717 10.1159/000081340

[50] Yao DX Flieder DB Hoda SA. Pulmonary tumor thrombotic microangiopathy: an often missed antemortem diagnosis. Arch Pathol Lab Med 2001;125:304–5. 11175660 10.5858/2001-125-0304-PTTM

[51] Lo AW Tse GM Chu WC Chan ABW. Pulmonary tumour microembolism clinically mimicking alveolitis. J Clin Pathol 2003;56:866–7. 14600135 10.1136/jcp.56.11.866 PMC1770106

[52] Lambert-Jensen P Mertz H Nyvad O Gong LD Cheng TO. Subacute cor pulmonale due to microscopic pulmonary tumour cell embolization. J Intern Med 1994;236:597–8. 7964440 10.1111/j.1365-2796.1994.tb00852.x

[53] Torbicki A Perrier A Konstantinides S. Guidelines on the diagnosis and management of acute pulmonary embolism: the Task Force for the Diagnosis and Management of Acute Pulmonary Embolism of the European Society of Cardiology (ESC). Eur Heart J 2008;29:2276–315. 18757870 10.1093/eurheartj/ehn310

[54] Qanadli SD Hajjam ME Mesurolle B. Pulmonary embolism detection: prospective evaluation of dual-section helical CT versus selective pulmonary arteriography in 157 patients. Radiology 2000;217:447–55. 11058644 10.1148/radiology.217.2.r00nv01447

[55] Goldhaber SZ. Pulmonary embolism. Lancet 2004;363:1295–305. 15094276 10.1016/S0140-6736(04)16004-2

[56] Serena G Gonzalez J Gaynor JJ Salerno T Verzaro R Ciancio G. Pulmonary tumor embolization as early manifestation in patients with renal cell carcinoma and tumor thrombus: perioperative management and outcomes. J Card Surg 2019;34:1018–23. 31376225 10.1111/jocs.14182

[57] Shin DS Ingraham CR Dighe MK. Surgical resection of a malignant liver lesion: what the surgeon wants the radiologist to know. AJR Am J Roentgenol 2014;203:W21–33. 24951226 10.2214/AJR.13.11701

[58] Bhargava P Iyer RS Moshiri M. Radiologic-pathologic correlation of uncommon mesenchymal liver tumors. Curr Probl Diagn Radiol 2013;42:183–90. 24070712 10.1067/j.cpradiol.2013.05.002

[59] Fowler KJ Sheybani A Parker RA 3rd. Combined hepatocellular and cholangiocarcinoma (biphenotypic) tumors: imaging features and diagnostic accuracy of contrast-enhanced CT and MRI. AJR Am J Roentgenol 2013;201:332–9. 23883213 10.2214/AJR.12.9488

[60] Lyman GH Bohlke K Khorana AA. Venous thromboembolism prophylaxis and treatment in patients with cancer: American Society of Clinical Oncology clinical practice guideline update 2014. J Clin Oncol 2015;33:654–6. 25605844 10.1200/JCO.2014.59.7351 PMC4881372

[61] Farge D Frere C Connors JM. 2019 international clinical practice guidelines for the treatment and prophylaxis of venous thromboembolism in patients with cancer. Lancet Oncol 2019;20:e566–81. 31492632 10.1016/S1470-2045(19)30336-5

[62] Posch F Konigsbrugge O Zielinski C Pabinger I Ay C. Treatment of venous thromboembolism in patients with cancer: a network meta-analysis comparing efficacy and safety of anticoagulants. Thromb Res 2015;136:582–9. 26210891 10.1016/j.thromres.2015.07.011 PMC7311195

